# A Practical Treatise on Diseases of the Ear

**Published:** 1873-12

**Authors:** 


*A Practical Treatise on the Diseases of the Ear, including the Analomy of the Organ.* By Dr. B. St. John Roosa, M. A., M. D:, Prof. of Diseases of the Eye and Ear in the University of the city of New York, &c., &c. New York: Wm. Wood & Co., D. B; Buffalo: H. H. Otis.

We know of no recent work upon Diseases of the Ear in the English language which has impressed us so favorably as has the work of Prof. Roosa. The author's large practical experience in the treatment of diseases of the ear, together with his knowledge of the literature of the subject, render him eminently able to produce a treatise which will be at once received as authoritative upon the subject.

The volume opens with a history of the progress of otology from the earliest ages of the profession down to the present time. This concludes with a list of authorities consulted, which will serve admirably as reference to those who wish to persue the subject still further.

Part first treats of the external, second of the middle, and third of the internal ear. Part fourth considers deaf mutism and hearing trumpets. The anatomy of the parts is founded on the description of Henle. The diseases of each part of the ear are well considered, the history of the different modes of treatment which have been persued occupying a portion of each department. The chapters close with a list of works upon otology which will be consulted with interest by those interested in the bibliography of the subject.

The work is amply illustrated, and forms for students and practititioners one of the best text books of which we have any knowledge. Were all writers so careful in their statements and so thorough in their investigations, we should not find so many contradictory statements in our standard works.

The little book by Dr. Dalby upon Diseases and Injuries of the Ear, comprises a series of eleven lectures upon the important points in otology delivered in St. George's Hospital. They do not pretent to be a scientific exposition of the subject, but simply a brief review of the more important points which might arise in a consideration of diseases and injuries of the ear Both volumes will find their proper place in the estimation of the profession, and we doubt not that each in its particular sphere will be found to fulfill all that can be desired of them. In regard to the first the American profession have cause to congratulate themselves that one of their number has produced so admirable a treatise upon a subject which a comparatively short time ago was but little understood, that little being confined to a few specialists in Europe.

